# Discerning the functional networks behind processing of music and speech through human vocalizations

**DOI:** 10.1371/journal.pone.0222796

**Published:** 2019-10-10

**Authors:** Arafat Angulo-Perkins, Luis Concha

**Affiliations:** 1 Instituto de Neurobiología, Universidad Nacional Autónoma de México, Querétaro, Querétaro, México; 2 Department of Cognitive Biology, Faculty of Life Sciences, University of Vienna, Vienna, Austria; 3 International Laboratory for Brain, Music and Sound (BRAMS), Montreal, Québec, Canada; Anadolu University, TURKEY

## Abstract

A fundamental question regarding music processing is its degree of independence from speech processing, in terms of their underlying neuroanatomy and influence of cognitive traits and abilities. Although a straight answer to that question is still lacking, a large number of studies have described where in the brain and in which contexts (tasks, stimuli, populations) this independence is, or is not, observed. We examined the independence between music and speech processing using functional magnetic resonance imagining and a stimulation paradigm with different human vocal sounds produced by the same voice. The stimuli were grouped as **Speech** (spoken sentences), **Hum** (hummed melodies), and **Song** (sung sentences); the sentences used in **Speech** and **Song** categories were the same, as well as the melodies used in the two musical categories. Each category had a scrambled counterpart which allowed us to render speech and melody unintelligible, while preserving global amplitude and frequency characteristics. Finally, we included a group of musicians to evaluate the influence of musical expertise. Similar global patterns of cortical activity were related to all sound categories compared to baseline, but important differences were evident. Regions more sensitive to musical sounds were located bilaterally in the anterior and posterior superior temporal gyrus (*planum polare* and *temporale*), the right supplementary and premotor areas, and the inferior frontal gyrus. However, only temporal areas and supplementary motor cortex remained music-selective after subtracting brain activity related to the scrambled stimuli. Speech-selective regions mainly affected by intelligibility of the stimuli were observed on the left *pars opecularis* and the anterior portion of the medial temporal gyrus. We did not find differences between musicians and non-musicians Our results confirmed music-selective cortical regions in associative cortices, independent of previous musical training.

## Introduction

Despite continuous debate regarding the level of functional, anatomical and cognitive independence between speech and music processing [1–5], it is clear that there are several differences regarding their basic acoustic properties (e.g., temporal, spectral or envelope features) and more importantly, they do not carry the same type of information (e.g., verbal aspects such as propositional meaning present in speech in comparison to music) [5–15]. Musical cognitive traits such as melodic, harmonic, timbral or rhythmic processing rely on basic analysis such as relative pitch, beat perception or metrical encoding of rhythm [13,16]. Interestingly, cortical activity (as measured through blood oxygenation level-dependent [BOLD] signal) is higher in secondary auditory cortices while listening to music as opposed to various types of non-musical human vocalizations—despite these regions being essential for speech processing [16,17]. The primary auditory cortex (i.e., Heschl’s gyrus), on the other hand, does not show differential activation towards these two types of acoustic categories [18–22]. These results suggest that while music and speech processing share the same basic auditory pathway until the primary auditory cortex, different patterns of activation are observed in other brain regions, with some exhibiting hemisphere lateralization [19–24].

The immediate question when comparing music and speech refers to human vocalizations, given that they represent the most common sound in our environment, and the most ancient expression of music and language [25–29]. In addition to their evolutionary relevance, human vocalizations provide experimental advantages, as they allow researchers to test both acoustic signals at the same time (e.g., song), or to explore them separately (i.e., just linguistic or melodic information) while evaluating perception, discrimination, memory tasks, or vocal production [24,30–36]. Nevertheless, the level of independence between speech and vocal music reported in some of these studies is still difficult to evaluate, particularly because only few studies have included the three basic vocal expressions to compare music, speech and their combination (i.e., speech, hum and song) [8,24,33]. In addition, studies using passive listening paradigms are scarce [8,24,30,31,37], with most studies having subjects perform tasks while they listen to different stimuli, or requiring vocal production (overt and covert) [8,32,33,36,38].

Previous findings have shown that regardless of musical stimuli being human vocalizations (such as a syllabic hum or song) there are certain differences in brain activity when compared to that elicited by speech, particularly a right hemisphere lateralization for music processing which could be modulated depending on the content of lyrics or tasks involved [8,24,32,33,39].

Given this, our goal was to evaluate at which point in the hierarchy of the auditory pathway these two acoustic signals show divergent processing, and if these differences in brain activation are maintained after altering some of the basic acoustic properties, which are supposed to involve primary areas of the auditory cortex according to the hierarchical functional organization [40–42]. To achieve this, we designed an experimental paradigm including three vocal categories, speech stimuli, and two different forms of musical vocal sounds, namely one with lyrics (song) and one without (hum). To evaluate which regions are functionally more affected by the low-level acoustic manipulations, we included scrambled versions of each category (i.e., **Speech**, **Song** and **Hum**) affecting the intelligibility of the vocal sound but preserving the amplitude and spectral features. In the same way, we controlled for semantic content and included different sentences that were sung and spoken by a professional singer. Melodic content was the same for the hummed and sung stimuli.

Our main hypothesis was that music-selective regions would show increased activity during both musical categories, in comparison to speech regardless of: 1) being produced by the same instrument, 2) the presence or absence of lyrics (i.e., song and hum, respectively) and 3) musical expertise. In the same way, we predicted that regions mainly modulated by speech processing would not show differences in their activation in response to similar categories (i.e., **Song** and **Speech**) but would differ with respect to the music category without verbal content (**Hum**). Based on previous results and numerous studies showing brain differences (anatomical and functional) in musicians as a result of training [19,43–50], we also hypothesized that musical training modulates cortical activity related to the three acoustic stimuli used. We therefore explored functional differences associated to musical training during vocal music perception between musicians and non-musicians.

## Results

**Fig 1:**
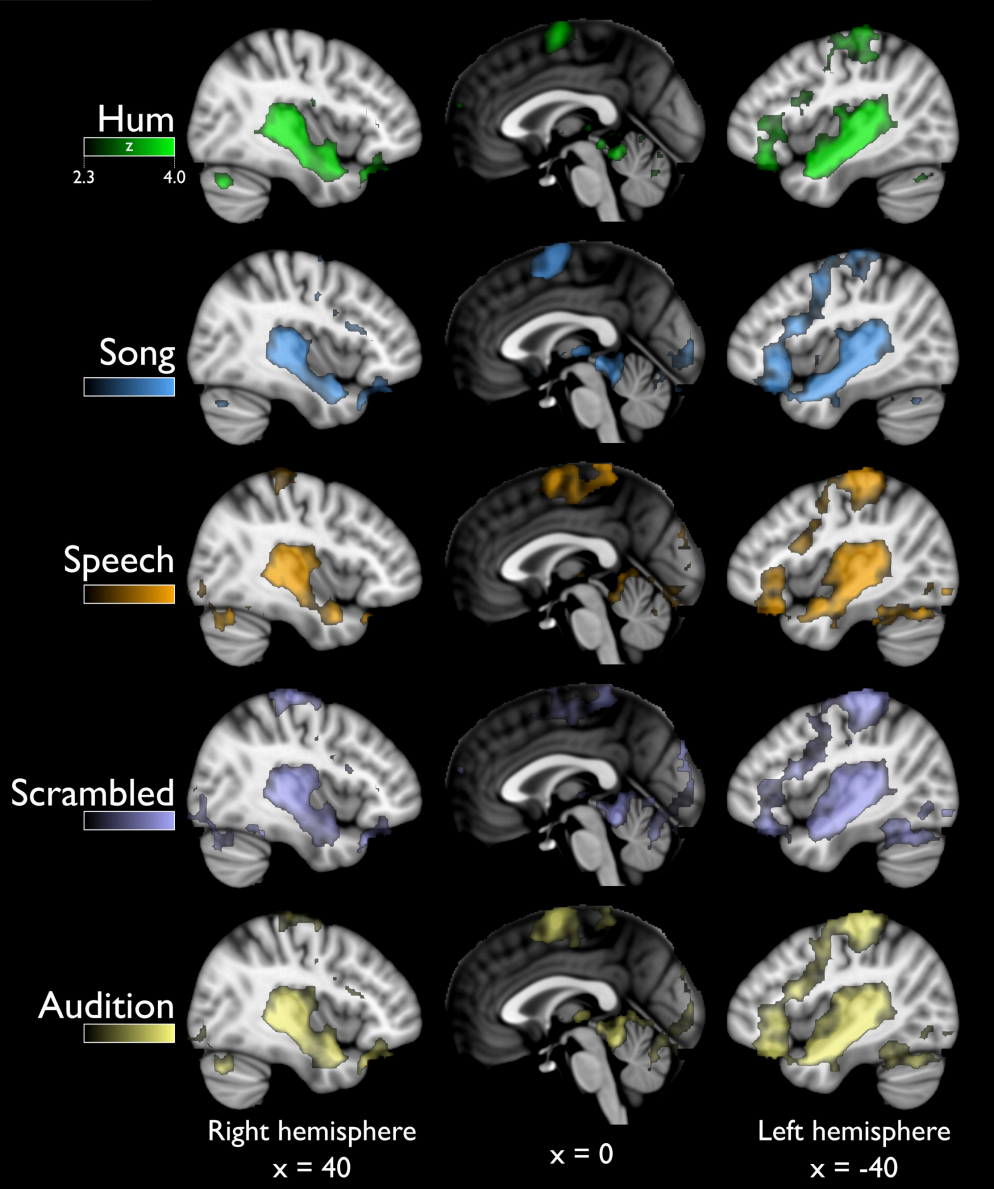
Global activity (above baseline) for all stimuli. Sagittal views showing distributed activation in response to all sound categories, greater than to scanner noise. The last row (Audition) includes all the previous categories. R = right, L = left. Statistical maps are overlaid on the MNI-152 atlas (coordinates shown in mm). For all categories color scale corresponds to z values in the range (2.3–4.0), as in top panel.

All sound categories produced BOLD signals that were significantly higher than those obtained during the baseline condition (i.e., scanner noise; Fig 1). Analysis of trigger responses to target sounds showed that all subjects responded appropriately (i.e., they were attentive to the stimuli).

### Analysis 1: Natural versus scrambled stimuli

**Fig 2:**
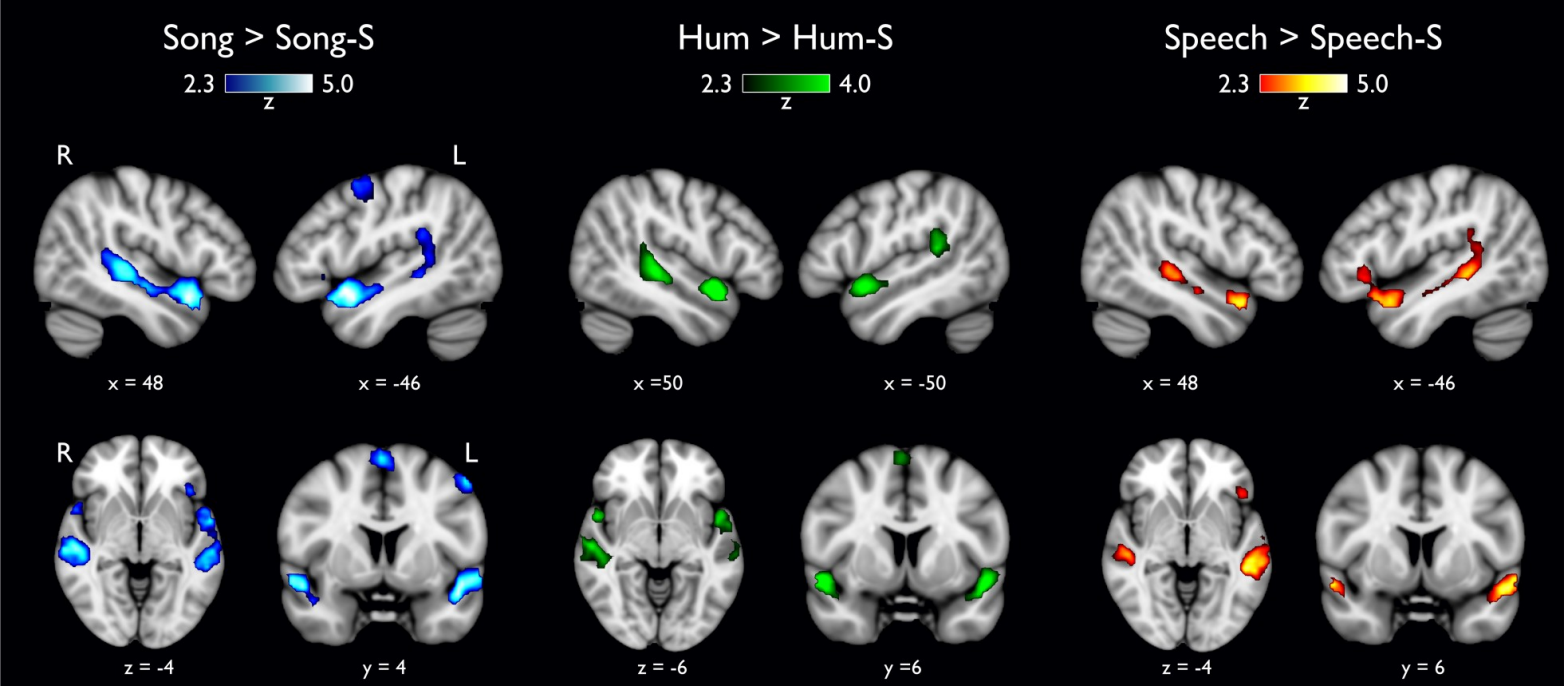
Natural stimuli versus scrambled stimuli. Each category contrasted against its own scrambled version (-S). No differences were found in Heschl’s gyrus; activation in the anterior portion of the superior temporal gyrus, specifically the *planum polare*, was found only in the musical categories (white arrowheads).

We searched for regions sensitive to spectral envelope modifications. All natural stimuli, when compared to the their scrambled counterparts, elicited stronger BOLD activity around (but not in) Heschl's gyrus in the superior temporal gyrus (STG). However, particular areas were differentially activated by specific categories, as outlined below and visualized in Fig 2. Statistical maps for this and all other analyses are available at https://neurovault.org/collections/DMKGWLFE/.

**1.1 Song versus Song-S.** The **Song > Song-S** comparison revealed 4 different clusters, two of them occupying the entire lateral aspect of the STG and the superior temporal sulcus (STS) bilaterally, with the notable exception of Heschl's gyrus; however, the most significant activation was located within the temporal pole (aSTG; blue colors in Fig 2). The left STG cluster extended into the inferior frontal gyrus (IFG) approximately in Brodmann areas 44 and 45. The third cluster had its most significant voxel in the right supplementary motor cortex (SMA) but it also covered the left counterpart, where it reached the dorsal premotor cortex (PMC). The opposite contrast (**Song-S > Song**) showed 4 clusters covering different regions: bilateral activation of primary auditory regions (Heschl's gyrus), the insular cortex and two bilateral activation of the middle and inferior occipital gyrus.

**1.2 Hum versus Hum-S.** The contrast testing for **Hum > Hum-S** showed bilateral activation along the STG with three peaks of maximal activation (Fig 2, green colors), two of which corresponded to the left and right temporal pole and the other one to the *planum temporale*. The fourth cluster was located in the supplementary motor area (SMA). No differences were found in Heschl's gyrus. The inverse contrast testing for **Hum-S > Hum** evidenced two clusters corresponding to Heschl's gyrus occupying the adjacent lateral face of the STG.

**1.3 Speech versus Speech-S.** Functional maps testing for **Speech > Speech-S** showed two large clusters distributed along the ventral lateral face of the STG reaching the dorsal area of the STS, in the left hemisphere the cluster presented a much larger volume (left 17,824 mm^3^ and right 5,552 mm^3^; Fig 2, warm colors). The left cluster included the IFG, as did the **Song** > **Song-S** contrast. The analysis of the opposite comparison (**Speech-S > Speech**) also revealed two clusters located in the lateral aspect of Heschl's gyrus (in each hemisphere); cluster locations were almost identical to those found in **Hum-S > Hum** and in **Song-S** > **Song**.

### Analysis 2: Activation patterns for Song versus Speech

**Fig 3:**
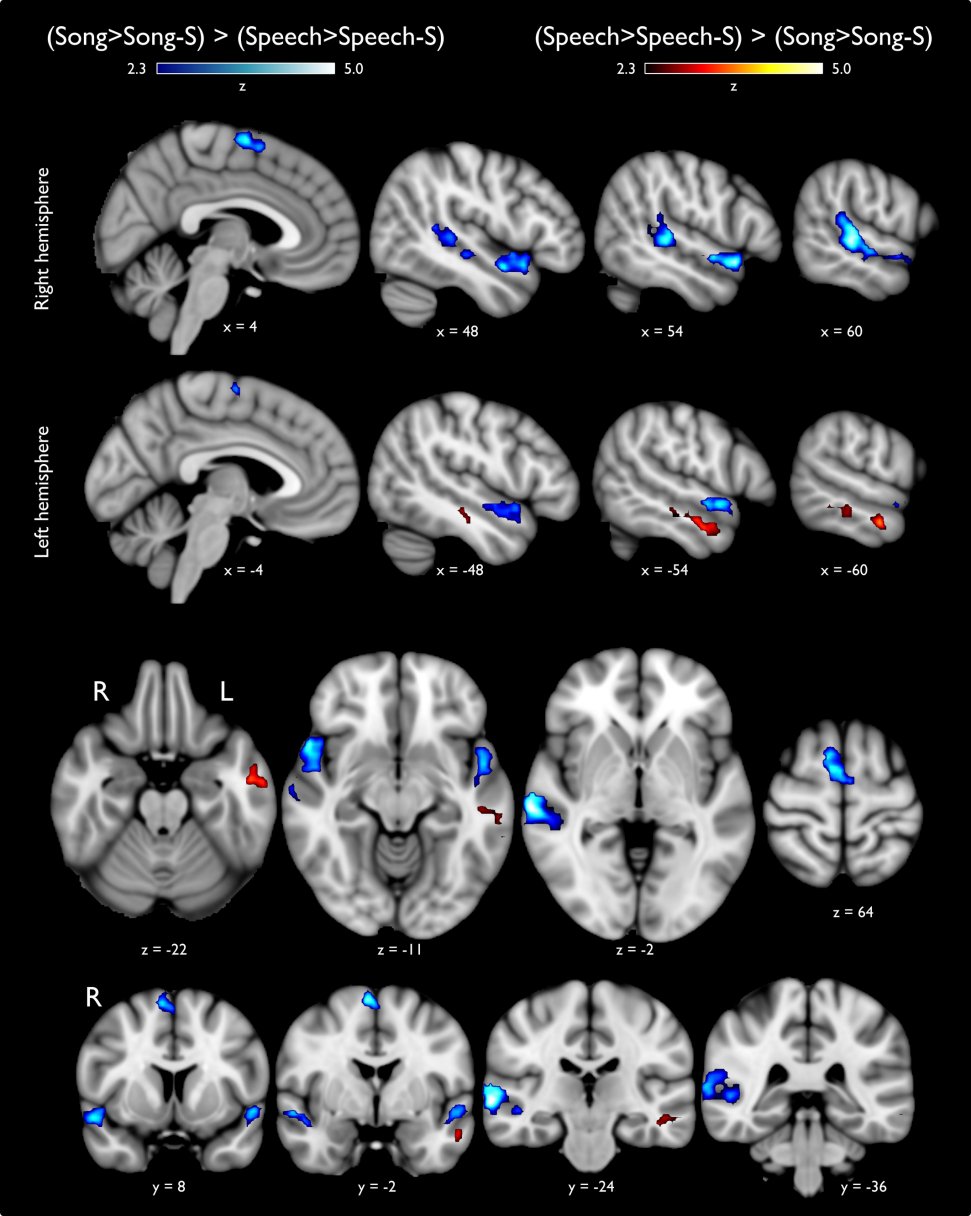
Statistical activation maps comparing vocal music and speech sounds. Statistical activation maps for differences between music and speech sounds after subtraction of activity elicited by their scrambled versions (-S). Blue colors indicate those regions that were more active while listening to song stimuli as compared to speech; warm colors show the opposite comparison. Upper and middle panels show lateral progression in the sagittal plane, from medial to lateral regions in each hemisphere. Axial and coronal views are presented in the inferior two panels to facilitate visual inspection.

To search for differences between **Song** and **Speech** categories that are not explained solely by differences in their acoustic features, we performed a high-level statistical analysis comparing the parameter estimates derived from Analysis 1. The contrast (**Song>Song-S)** > (**Speech>Speech-S)** generated three different clusters. Two clusters were distributed along the right STG from the *planum temporale* to the *planum polare*, sparing Heschl's gyrus (Fig 3, cold colors); in the left hemisphere the activation was located on the aSTG covering part of the *planum polare*. Another cluster was located over the right supplementary motor area (SMA). The opposite contrast ([**Speech>Speech-S]** > [**Song>Song-S]**; Fig 3, warm colors) elicited focal activation located on the anterior portion of the right middle temporal gyrus (MTG) cortex, extending slightly into the superior temporal sulcus (STS). Differences between categories without controlling for their scrambled counterparts are shown in S1 Fig, which also extends Analyses 3 and 4.

### Analysis 3: Activation patterns for Hum versus Speech

By excluding lyrics and leaving melodic information intact (i.e., **Hum**), we aimed to observe music-sensitive regions when compared with activations elicited by **Speech**. Similarly to Analysis 2, the contribution of acoustic features to brain activiy was controlled by subtracting parameter estimates related to the corresponding scrambled acoustic categories.

**Fig 4:**
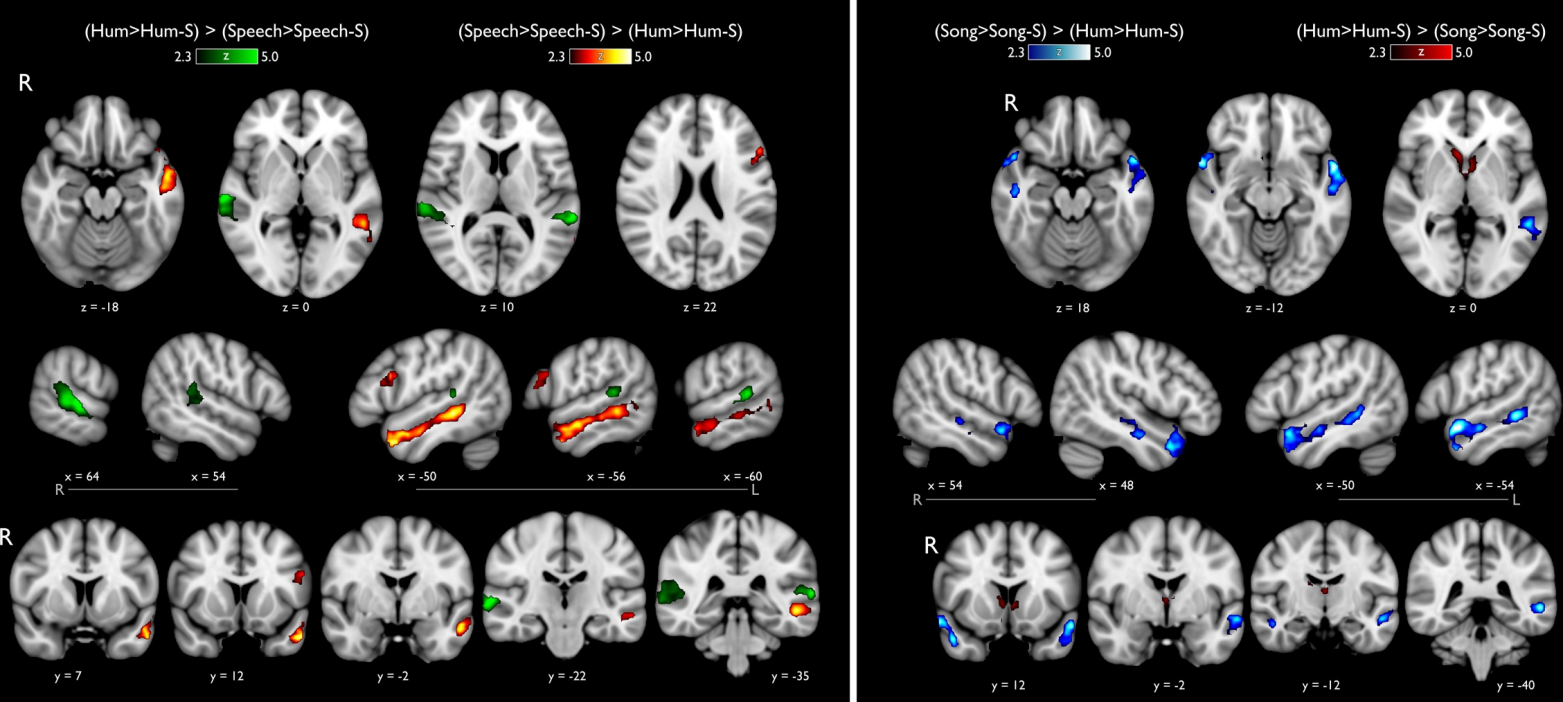
Statistical activation maps for Hum versus Song and Speech. Left panel: Hum *versus* Speech, after subtraction of activity elicited by their scrambled counterparts (-S). Listening to hummed songs elicited enhanced activity of the *planum temporale* bilaterally, as compared to that elicited by speech stimuli (green colors). The opposite contrast (warm colors) shows activation only in the left hemisphere including the IFG, MTG and STS. Right panel: Vocal-music with and without lyrics. Lateral, axial and coronal views showing in blue the cortical regions modulated preferentially to Song as compared to Hum stimuli, after removing the effect of their scrambled counterparts. Activation extended bilaterally over the aSTG and STS. The opposite contrast showed activation on the caudate nucleus.

Functional maps testing for (**Hum>Hum-S) > (Speech>Speech-S)** (Fig 4; left panel, green colors) revealed bilateral activation on the posterior STG (pSTG). In both hemispheres the activation was observed on the *planum temporale*. The opposite comparison ([**Speech>Speech-S] > [Hum>Hum-S]**) showed significant activity distributed along the left STS and dorsal MTG (Fig 4, left panel, warm colors), the cluster extended from the posterior regions until the anterior portion of the MTG (aMTG). A second cluster was located on the *pars opecularis* from the left inferior frontal gyrus (IFG).

### Analysis 4: Comparing vocal-music with and without lyrics

This analysis compared the brain activation associated to **Song** stimuli (i.e., music with lyrics) with the hummed version, as a way to explore the question regarding sharing resources between music and speech. The results obtained from the contrast (**Song>Song-S)** > (**Hum>Hum-S)** (Fig 4; right panel, cold colors) showed activation covering the anterior and posterior aspects of the left STS, the cluster reached the dorsal portion of the MTG and the anterior portion and the most anterior portion of the STG (aSTG). In the right hemisphere we found the middle portion of the STS and the newly the aSTG. The reverse contrast ([**Hum>Hum-S]** > [**Song>Song-S]**) showed the caudate nucleus, bilaterally.

### Analysis 5: Influence of musical training

We evaluated whether musical training differentially modulated brain activity while listening to our experimental paradigm. All the contrasts described in Analyses 2–4 were tested for group differences, with none being statistically significant.

### Analysis 6: Functional characterization of the superior temporal gyrus

**Fig 5:**
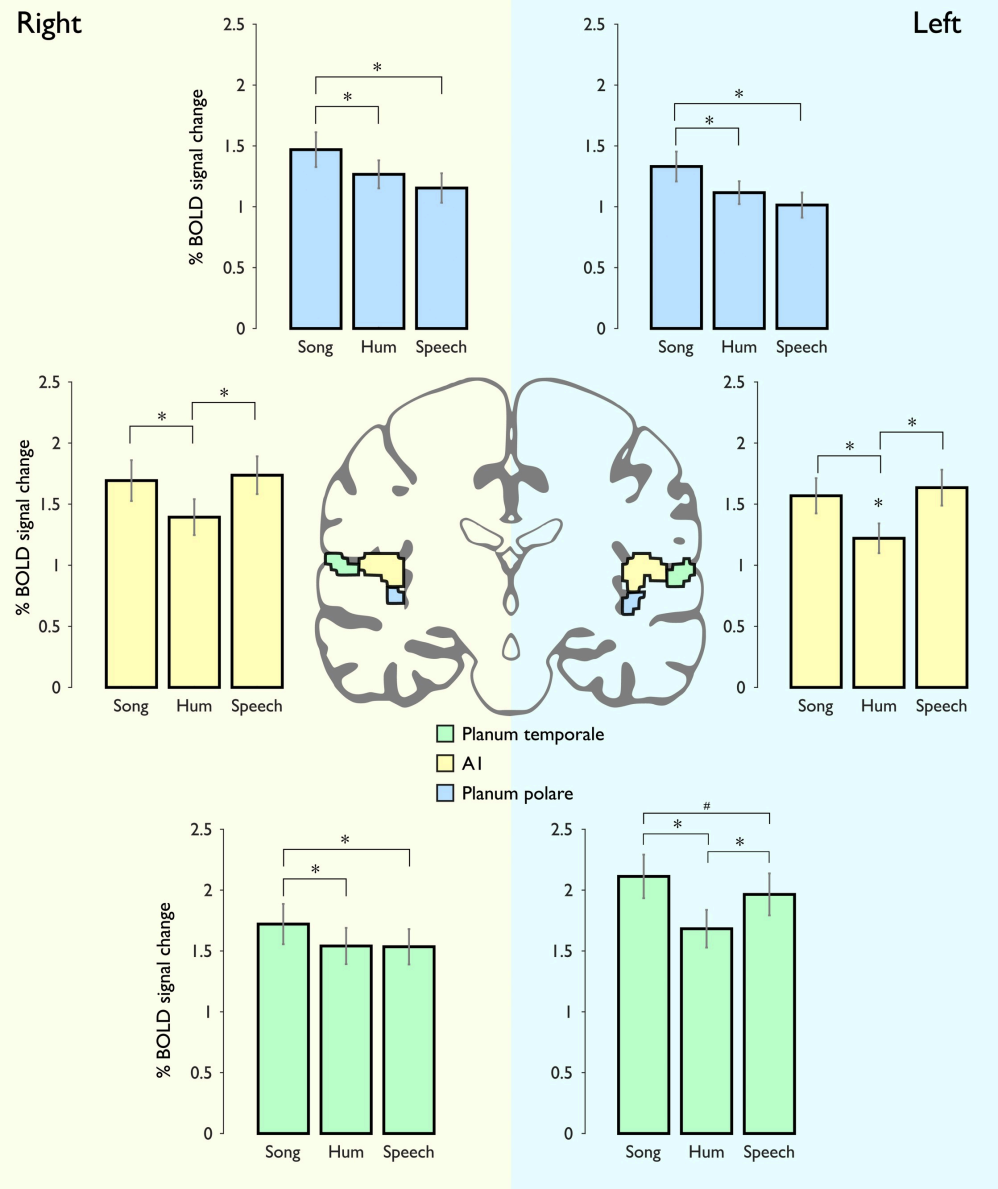
ROIs of Heschl's gyrus, planum polare and planum temporale. Right and left hemispheres are shaded in different colors. Top panel: The percentage of BOLD signal modulation in the *planum polare* (blue color) showed similar patterns between the right and left hemisphere, the highest level of activation in this region corresponded to Song, followed by Hum and Speech sounds; statistical differences were found between Song and Speech. Middle panel: BOLD signal changes in the right and left Heschl's gyrus (yellow color), no differences between Song and Speech stimuli. Bottom panel: The left and right *planum temporale* (green color) exhibited different patterns of activation, in the right hemisphere Song stimuli elicited the highest values, while in the left hemisphere there were no differences between them. * indicates significant difference from the rest of the stimuli (p<0.0028; Bonferroni correction); # stands for uncorrected p<0.05.

To further analyze BOLD signal changes derived from the different sound categories along the auditory cortices we used an unbiased approach to compare BOLD changes in the *planum polare*, *planum temporale* and Heschl’s gyrus. Fig 5 illustrates the percentage of BOLD signal change for each category (significance levels = * p < 0.0028; **#** p < 0.05). We found statistically significant differences between **Song** and **Speech** in the left and right *planum polare*, and also between **Song** and **Hum**; the lowest levels of activation within the *planum polare* were observed in the **Speech** category. BOLD responses in Heschl's gyrus (A1) were lower in response to **Hum** as compared to those elicited by **Song** or **Speech**, bilaterally; there were no differences in A1 activation between the last two categories. In parallel with this observation, acoustic feature analyses showed that **Hum** differs substantially from **Song**/**Speech** in several aspects, such as zero-cross, and spectral spread, brightness, centroid, kurtosis and flatness (S2 Fig).

The *planum temporale* showed different patterns between hemispheres. The right *planum temporale* was equally active during **Hum** and **Speech** conditions in contrast to **Song**; however, the left *planum temporale* exhibited similar pattern to that seen in A1, namely it was preferentially activated by **Song** and **Speech** categories (Fig 5; lower panel). From all comparisons among the three different regions in the STG, the highest activation were found in the left *planum temporale*.

## Discussion

In this study we aimed to further explore brain regions modulated for music as compared to speech. In Particular, we investigated whether this selectivity changed when we combined the two domains (vocal music with lyrics), by comparing the **Song** category against **Speech**, or **Hum,** while we controlled for differences of timbre (using the same voice to produce all the stimuli), melodic, and semantic content (using the same sentences and same musical excerpts).

To identify brain regions sensitive to musical sounds at different processing levels, we first compared each natural vocal category against their scrambled versions. We observed that while all acoustic categories recruited different regions of the STG when compared them to their scrambled version, only the musical categories also revealed activation maps covering the SMA and PMC. This result confirms what has been previously described in speech processing showing that music is also hierarchically encoded involving brain regions beyond Heschl’s gyrus [19,20,51,52]. All acoustic categories induced activations of the left posterior Sylvian region at the parietal-temporal junction (Spt), which has been suggested as a region integrating audio motor patterns from the vocal tract [53,54]. The contrast testing for **Song > Song-S** revealed bilateral activation of the *planum polare* and *temporale*, left PMC and SMA. This result suggests these areas are not exclusively modulated by low-level acoustic parameters, but also by the temporal structure affecting the semantic, syntactic and melodic-contour, as has been reported in other musical studies [22,55,56].

The opposite contrasts comparing the scrambled versions (e.g., **Song-S > Song**) revealed consistent higher BOLD modulations in Heschl’s gyrus. The increased activity in response to scrambled acoustic stimuli, independently of acoustic category, is indicative of converging processing of music and speech processing at this level. The primary auditory cortex presents a tonotopical organization sensitive to frequency tuning and spectro-temporal modulations [57–61], which are present in all the categories we used. However, the primary auditory cortex also contributes to the construction of auditory objects, and it has been suggested that less meaningful sounds require more neural resources to extract information from them, in an attempt to elaborate predictions about their possible identity as an auditory object [60,62].

Once we had identified the brain regions that are sensitive to the temporal structure of acoustic stimuli, we searched for the functional independence between music and speech processing. The contrast (**Song>Song-S) >** (**Speech>Speech-S)** (Fig 3) showed that the *planum polare* bilaterally, the right *planum temporale*, and SMA, are auditory regions particularly relevant for music processing in comparison to speech, independently of the similarities regarding semantic content. In addition, results from contrast comparing (**Hum>Hum-S) >** (**Speech>Speech-S)** (Fig 4), highlight the functional identity of the *planum temporale*, as it was the only region showing consistently increased activity in response to musical conditions. The *planum polare*, which is very active bilaterally in the **Song** condition (as compared to activity elicited by either **Speech** or **Hum**) was not more responsive to **Hum** as it was to **Speech**. Thus, the *planum polare* showed higher levels of activation in both musical conditions, particularly when intelligibility was not affected. However, stronger bilateral activation was observed when vocal music included lyrics (e.g., [**Song>Song-S] > [Hum>Hum-S]**), likely reflecting the simultaneous processing of speech and music present in **Song**. This finding was corroborated with an unbiased ROI analysis (Fig 5). In line with previous reports, our results suggest the *planum polare* may play an intermediate role between the primary auditory cortex and other associative cortices, possibly extracting information such as melodic patterns or pitch-interval ratios, required for further processing leading to perceptual evaluation of complex musical patterns [34,63]. The *planum polare* has been specifically reported in other studies evaluating vocal music while performing a same-different task [8,24] and during in over and cover vocal production tasks [32,33,64]. Similarly, the largest activation of the right *planum temporale* was elicited by **Song** stimuli, (whereas no significant differences were found between **Hum** and **Speech)**, suggesting it has a shared functional identity during vocal music and speech processing, specially when they are combined (**Song**). On the other hand, the *left planum temporale* was equally modulated by **Speech** and **Song** categories, confirming its preference for stimuli with verbal content (whether musical or not), possibly associated with representations of lexical or semantic structures [51,65,66].

Our results showed music-sensitivity in cortical motor regions such as the SMA and the PMC, which have been related to beat and rhythm perception [67–72]. Particularly, SMA showed music preference independently of whether the music was hummed or sung and also regardless of musical training. These results support a particular role of SMA in music processing and its relation to action, as Lima and colleagues [73] suggested a possible role in motor facilitation to prepare and organize movement sequences.

It has been described that speech processing involves distributed cortical regions (e.g., primary auditory cortices, left STG, MTG, *planum temporale*, STS, IFG, post-central gyrus and the ventral division of M1) [74,75], which are recapitulated in our results (Fig 2 and S1 Fig). While **Speech** elicited larger activity than **Hum** in the entire left MTG (Fig 4), only the most anterior aspect of MTG was more sensitive to **Speech** than **Song** (Fig 3), suggesting a predominant role of the MTG in the processing of linguistic content.

In a previous study, we found differences between musicians and non-musicians involving greater bilateral activity of the *planum polare* and the right *planum temporale*, when listening to different types of instrumental music stimuli in comparison to speech [19]. In our current work, however, we did not find evidence that musical expertise modulates brain regions while processing vocal music or speech. We suggest this negative finding is indicative of the common exposure to (and perhaps similar production of) vocal music, as singing is commonplace in everyday situations since early age (e.g., birthdays, hymns, cheers); contrarily, learning to play an instrument is not a universal activity. Nonetheless, other factors may be responsible for the discrepancy with our previous report, such as the relatively smaller size of our current sample (17 musicians), and the diversity of musical training in the group of musicians (S1 Table), which may induce functional plastic changes related to instrument-specific tuning [49,50]. The effect of musical training should therefore be explored further and more thoroughly (e.g., by studying professional singers).

### Limitations of the Study

Together, these data demonstrate different cortical regions that are preferentially modulated by particular sounds, whether they are music, speech or a mixture of the two. However, the anatomical resolution given by the technique does not allow us to distinguish finer anatomical details regarding the specific distribution of the statistical maps. For the same reason, we can not elaborate a more detailed analysis regarding the participation of the primary auditory cortex in **Speech** and S**ong** conditions, for example. Temporal resolution is also limited in all fMRI studies, and there is a wealth of information from rapid temporal fluctuations present in music and language that are not easily addressed with this technique. As such, our results can only reveal relatively long-term changes of brain activity in response to listening to specific sound categories, and our conclusions will benefit greatly from other methods with high temporal resolution, such as (magneto-) electrophysiological recordings. Finally, although stimuli of all three categories were produced by the same singer, we acknowledge that other acoustic features were not homogenized to control for all acoustic parameters that could potentially differ between categories. While some differences in cortical activity may be explained by these low-level features, our main findings are better explained by higher-level, time-varying acoustic features that are characteristic of each category.

## Conclusions

Our data indicates that music selectivity in distributed brain regions independently of using vocal music with and without lyrics. Music-sensitive regions involved frontal and temporal cortical areas, specially in the right hemisphere. Our results indicate that the temporal structure of vocal music and speech is processed in a large temporal-frontal network, and that speech and music processing diverge, with specific regions (such as the *planum polare* and *temporale*, and SMA), being particularly sensitive to the temporal structure and acoustical properties of music subjected to further processing beyond the primary auditory cortices.

## Methods

### Participants

Thirty-three healthy, right-handed volunteers, age 28 ± 8 years (range: 20 to 42 years; 17 women), participated in this study. Seventeen volunteers were non-musicians (age 27 ± 6 years; range: 20–45 years; 9 women), who had not received extra-curricular music education beyond mandatory courses in school. Musicians (16 volunteers; age 28 ± 7 years; range: 20–42 years; 8 women) had received at least 3 years of formal training/studies in music (either instruments or singing) and were currently involved in musical activities on a daily basis (S1 Table). Groups did not differ in terms of age or gender. All volunteers were native Spanish speakers, self-reported normal hearing (which was confirmed during an audio test within the scanner), were free of contraindications for MRI scanning and gave written informed consent before the scanning session. The research protocol had approval from the Ethics Committee of the Institute of Neurobiology at the Universidad Nacional Autónoma de México and was conducted in accordance with the international standards of the Declaration of Helsinki of 1964.

### Experimental design

**Fig 6:**
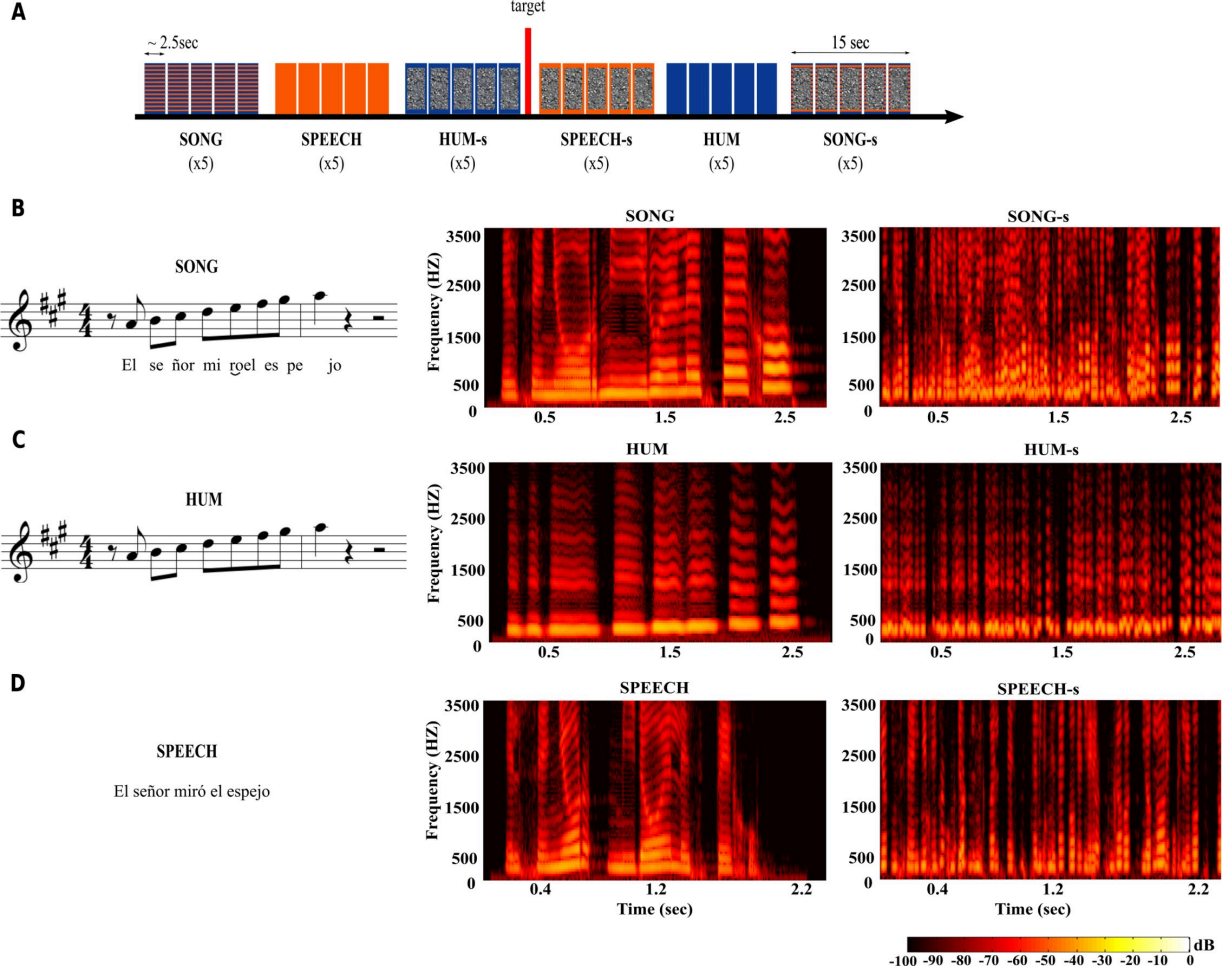
Auditory stimulation paradigm. Each block (~15 sec) included 5–7 different stimuli from the same category (A). The paradigm included 5 blocks of each of the 6 sound categories, for a total duration of 10 min. Blue indicates musical sounds, orange for speech stimuli, and scrambled gray indicates the scrambled version of each category (e.g., Hum-S). The target sound was presented between two randomly selected blocks on five occasions throughout the paradigm. (B-D) Example stimulus in three versions: Song (B), Hum (C) and Speech (D). All stimuli were produced by the same singer. Spectrograms show the frequency structure (y-axis) over time (x-axis), with colors representing the relative amplitude of each frequency band. Left column shows natural categories; right column shows the temporally scrambled condition. “El señor miró el espejo”: “The man looked at the mirror”.

The vocal music paradigm used a pseudo-randomized block design; each block lasted 15 seconds and included 5–7 stimuli from the same category (Fig 6, panel A). Six different categories were included: **Hum**, **Song**, **Speech**, and their scrambled counterparts (5 blocks each along the paradigm). Additionally, 5 blocks of silence (each lasting 15 seconds) were interspersed throughout the stimulation paradigm. The stimulation protocol had a total duration of ~10 minutes.

The stimulation paradigm was presented with E-prime Study Software (version 2.0; Psychology Software Tools, Sharpsburg, PA) binaurally through MRI-compatible headphones (Nordic NeuroLab, Bergen, Norway) that attenuated acoustic interference (~20 dB) generated by the gradients. While not used as a formal test for normal hearing, we performed a short audio test (1 min) inside the scanner, using similar stimuli, to evaluate whether volunteers could hear and recognize the different sounds inside the scanner; the volume was deemed comfortable but sufficiently high to mask the noise generated by the imaging acquisitions.

Subjects were instructed to pay attention to the stimuli and to press a button with the index finger of their right hand every time they heard a pure tone (500 Hz, 500 ms duration), which was presented 5 times randomly throughout the paradigm. We used this strategy to ensure attention throughout the stimulation paradigm, and we used a pure tone as it is clearly different from the rest of our stimuli and therefore easily recognizable. Subjects kept their eyes open during scanning.

### Acoustic stimuli

**Vocal-music paradigm** (Fig 6, panel A): Auditory stimuli consisted of short excerpts of 2.8 ± 0.5 seconds, normalized to -30 dB using Adobe Audition (Adobe Systems). MIRToolbox, implemented in MATLAB (Mathworks, Natick, MA)[76], was used for acoustic analysis.

All stimuli (divided into **Song**, **Speech** and **Hum** categories; Fig 6, panel B-D) were produced by a professional female singer after a period of training to avoid any emotional emphasis during production (e.g., affective prosody or emotional intonation). The sentences used in the **Song** and **Speech** categories were novel and carefully selected from a pool of 80 phrases in a pilot test, where 30 listeners (15 women), who did not participate in the main experiment, rated the emotional valence (e.g., from very emotional to neutral) and the complexity of each stimulus. Finally, we selected for use in the imaging experiments the 35 sentences considered the most neutral and simple, both in their grammatical structure and meaning (e.g., “La alfombra está en la sala”—“The rug is in the living room”).

1. **Hum:** This category included 25 novel musical sequences that we had previously used [19,77]; all melodies followed rules of Western tonal music. The singer hummed these melodies with her mouth closed (i.e., no syllable was used). Each block in this category consisted of five different melodies.
2. **Speech:** 35 Spanish sentences were included in this category. Given the slightly shorter duration of spoken sentences as compared to their sung versions, seven phrases were presented per block.
3. **Song:** The same 25 musical sequences from the **Hum** category were used as melodies to produce the sung versions of 25 sentences used in the speech condition. Five songs were included per block.

**Scrambled stimuli** (Fig 6, panel B-D): Scrambled versions for the three main categories. Small fragments (50 ms) with 50% overlap were randomly repositioned temporally within an interval of one second using a freely available Matlab toolbox (http://www.ee.columbia.edu/~dpwe/resources/matlab/scramble/). This procedure retained low level acoustical attributes (i.e., pitch, duration, loudness, and timbre) but rendered the stimuli unintelligible by disrupting their temporal organization (i.e., melody and rhythm) and therefore, their high-level perceptual and cognitive properties. The scrambled counterparts of each of the original sound categories are identified as: 4) **Hum-S,** 5) **Speech-S** and 6) **Song-S**.

### Image acquisition

All images were acquired at the National Laboratory for Magnetic Resonance Imaging using a 3T Discovery MR750 scanner (General Electric, Waukesha, Wisconsin) with a 32-channel coil. Functional volumes consisted of 50 slices (3 mm thick), acquired with a gradient-echo, echo-planar imaging sequence with the following parameters: field of view (FOV) = 256×256 mm^2^, matrix size = 128×128 (voxel size = 2×2×3 mm^3^), TR = 3000 ms, TE = 40 ms. To improve image registration we also acquired a 3D T1-weighted volume with the following characteristics: voxel size = 1×1×1 mm^3^, TR = 2.3 s, TE = 3 ms. The total duration of the experiment was 18 minutes. All imaging data are freely accessible at https://openneuro.org/datasets/ds001482.

### Image processing and statistical analyses

Anatomical and functional images were preprocessed using *fsl* tools (version 5.0.9, fMRIB, Oxford UK). Images were corrected for movement and smoothed using a 5-mm FWHM Gaussian kernel; spatial normalization was performed using the MNI-152 standard template as reference. fMRI data analysis was conducted using FEAT (FMRI Expert Analysis Tool) version 6.00. Statistical analysis was performed using the general linear model. For each subject (first level analysis) the three original acoustic categories were modeled as explanatory variables (EV), along with their three scrambled counterparts (Analysis 1). Target stimuli were included as a nuisance regressor. Statistical maps of between-category differences for each subject were generated using a fixed-effects model, and the resulting contrasts were entered in a random-effects model for between-subject analyses using FLAME [78,79]; musical expertise was included as a group factor at this level. We used random field theory [80] to correct for multiple comparisons (voxel z > 2.3, cluster p < 0.05) unless otherwise specified.

### Analyses

**Analysis 1 (natural *versus* scrambled stimuli):** By disrupting the global perception of the stimuli through temporal scrambling, while leaving low-level acoustic features untouched, we searched for brain areas that showed greater activation by natural stimuli compared to their scrambled counterparts in each category. The contrasts included were: 1.1 **Song** *vs*. **Song-S**; 1.2 **Hum** *vs*. **Hum-S** and 1.3 **Speech** *vs*. **Speech-S**.

**Analysis 2 (Song *versus* Speech):** We searched for differences in brain activity in response to listening to song and speech stimuli, both of which include semantic information but differ in temporal (e.g. rhythm) and spectral (e.g. pitch modulation) content. The contrasts included were: 2.1 **Song>Song-S** *vs* **Speech>Speech-S**; 2.2 **Speech>Speech-S** *vs*. **Song>Song-S**.

**Analysis 3 (Hum *versus* Speech)**: These two categories differ in terms of temporal, spectral and semantic content. The contrasts included were: 3.1 (**Hum>Hum-S)** *vs*. *(***Speech>Speech-S)**; 3.2 (**Speech>Speech-S** *vs*. (**Hum>Hum-S)**.

**Analysis 4 (Hum *versus* Song)**: This analysis aimed to find differences in brain activity in response to listening to two categories of melodic sounds that differed in semantic content. The contrasts included were: 4.1 (**Hum>Hum-S)** *vs*. (**Song>Song-S)**; 4.2 (**Song>Song-S)** *vs*. (**Hum>Hum-S)**.

**Analysis 5 (musicians *versus* non-musicians):** we evaluated differences between groups to identify whether musical training changes the patterns of activation during the perception of different types of human vocalizations. We included the following comparisons: 5.1 (**Song>Song-S)** *vs*. *(***Speech>Speech-S) |** musicians > non-musicians; 5.2 (**Speech>Speech-S)** *vs*. (**Song>Song-S) |** musicians > non-musicians; 5.3 (**Hum>Hum-S)** *vs*. *(***Speech>Speech-S) |** musicians > non-musicians; 5.4 (**Speech>Speech-S)** *vs*. (**Hum>Hum-S) |** musicians > non-musicians: 5.5 (**Song>Song-S)** *vs*. (**Hum>Hum-S) |** musicians > non-musicians; 5.6 (**Song** *vs*. **Hum) |** musicians > non-musicians.

**Analysis 6 (Functional characterization of the Superior Temporal Gyrus):** Finally, we also performed an independent ROI (region of interest) analysis of the *planum polare*, *planum temporale* and Heschl's gyrus. ROIs were derived from the Harvard-Oxford Probabilistic Anatomical Atlas thresholded at 33%; statistical significance threshold was set at p< 0.0028, considering eighteen comparisons were performed (i.e., three categories in six ROIs). This analysis explored BOLD signal modulations to melodic and non-melodic vocal sounds in three different auditory regions.

## Supporting information

S1 Fig Simple contrasts for the three original categories (Song, Hum and Speech). Left panel shows **Song** *vs* **Speech**; Middle panel **Hum** *vs* **Speech;** and Right panel shows shows **Song** *vs* **Hum**. Color codes are similar to Figs 2, 3 and 4. Statistical maps are overlaid on the MNI-152 atlas. MNI coordinates of each slice are expressed in mm.
(TIF)

S2 Fig Box plot of acoustic characteristics across categories. Horizontal bars represent the median; black boxes show the interquartile range; vertical lines show the data range excluding outliers (dots).
(TIF)

S1 Table Musical training information. (PDF)
