# Supplementary figures and images for: Discerning the functional networks behind processing of music and speech through human vocalizations
Source: PLoS One. 2019 Oct 10;14(10):e0222796. doi: 10.1371/journal.pone.0222796 (PMC6786620; doi:10.1371/journal.pone.0222796)

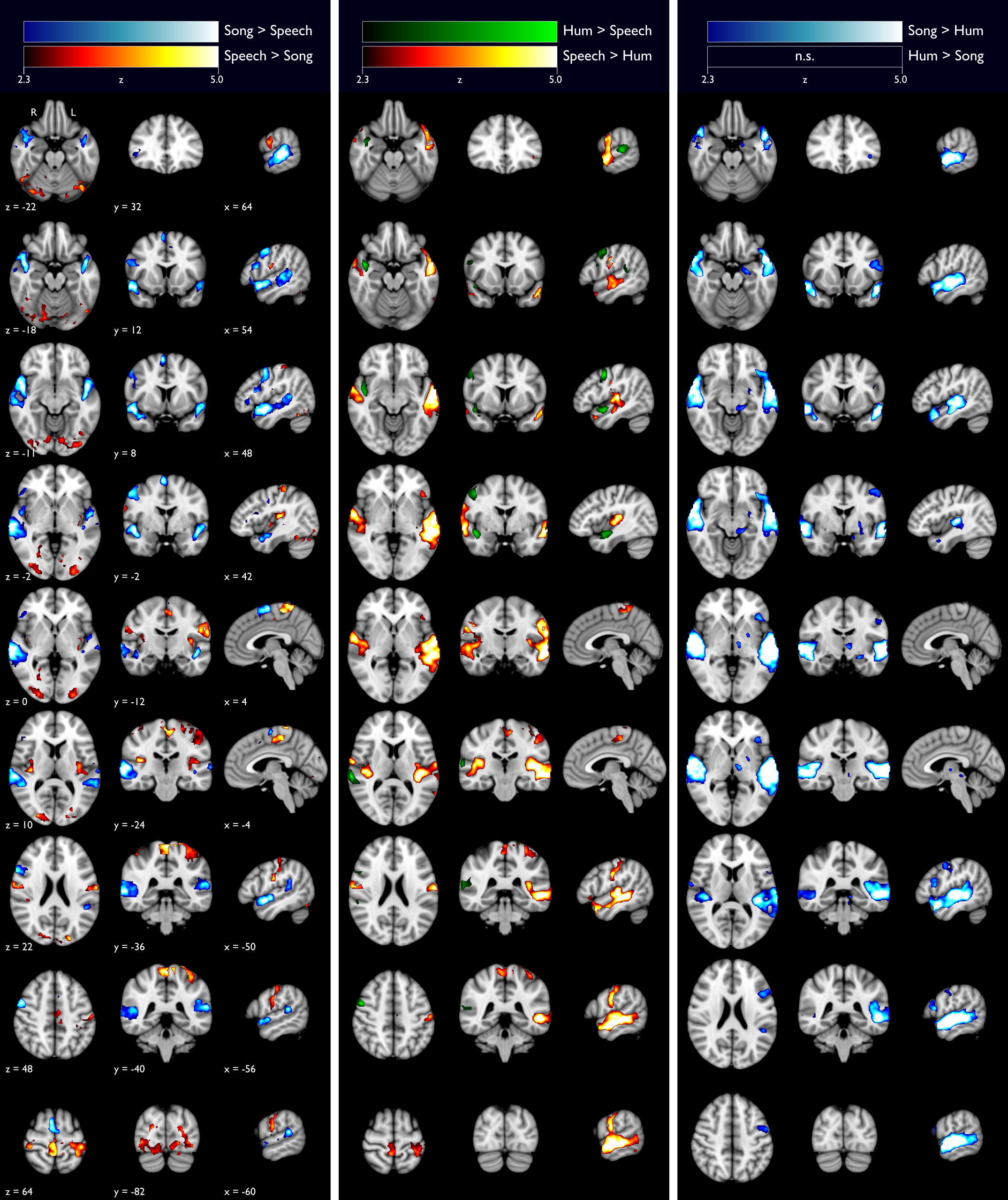

Supplement: S1 Fig — Left panel shows Song vs Speech; Middle panel Hum vs Speech; and Right panel shows shows Song vs Hum. Color codes are similar to Figs 2, 3 and 4. Statistical maps are overlaid on the MNI-152 atlas. MNI coordinates of each slice are expressed in mm. (TIF) [file pone.0222796.s001.tif]
