# Supplementary material for: Discerning the functional networks behind processing of music and speech through human vocalizations
Source: PLoS One. 2019 Oct 10;14(10):e0222796. doi: 10.1371/journal.pone.0222796 (PMC6786620; doi:10.1371/journal.pone.0222796)
Supplement: S1 Table — (PDF) [file pone.0222796.s003.pdf]

Suject	Practice (y)	Age of onset (y)	Weekly practice (h)	Weekly listening (h)	Instruments played	Main expertise
1	13	7	5	14	(5) guitar, keyboard, bass, harmonica and flute	(1) guitar
2	30	12	10	30	(3) flute, guitar, piano and violin	(1) violin
3	8	12	5	10	(7) piano, guitar, bass, ukulele, saxophone, synthesizer and percussions	(6) piano, guitar, bass, ukulele, saxophone and synthesizer
4	13	7	9	>30	(3) piano, psaltery, voice	(3) piano, psaltery, voice
5	8	18	15	50	(2) guitar and saxophone	(1)saxophone
6	12	12	12	14-20	(4) guitar, bass, mandoline and keyboard	(1) guitar
7	22	5	5	20	(4) piano, guitar, bass, harmonica	(2) guitar and piano
8	27	13	8	40	(2) guitar and violin	(1) guitar
9	21	6	15	50	(4) piano, guitar, bass, percussions	(2) piano and bass
10	13	8	32	50-60	(4) violin, piano, guitar, voice	(3) violin, piano and voice
11	19	7	20	28	(2) piano and voice	(1) voice
12	51	3	12	65	(3) piano, guitar and percussion	(1) guitar
13	7	18	18	12	(3) violin, paino and flute	(1) violin and piano
14	30	8	28	35-56	(1) piano	(1) piano
15	28	14	6	21-42	(3) voice, piano and guitar	(1) voice
16	2	29	3	10	(1) piano	(1) piano

Table S.1. Musical training information
